# The potential of advanced MR techniques for precision radiotherapy of glioblastoma

**DOI:** 10.1007/s10334-021-00997-y

**Published:** 2022-02-07

**Authors:** Patrick L. Y. Tang, Alejandra Méndez Romero, Jaap P. M. Jaspers, Esther A. H. Warnert

**Affiliations:** 1 Department of Radiotherapy, Erasmus MC Cancer Institute, University Medical Center, Rotterdam, The Netherlands; 2 Department of Radiology and Nuclear Medicine, Erasmus MC, University Medical Center, Rotterdam, The Netherlands

**Keywords:** Glioblastoma, Magnetic resonance imaging, Imaging biomarkers, Radiotherapy, Segmentation, Target volume delineation

## Abstract

As microscopic tumour infiltration of glioblastomas is not visible on conventional magnetic resonance (MR) imaging, an isotropic expansion of 1–2 cm around the visible tumour is applied to define the clinical target volume for radiotherapy. An opportunity to visualize microscopic infiltration arises with advanced MR imaging. In this review, various advanced MR biomarkers are explored that could improve target volume delineation for radiotherapy of glioblastomas. Various physiological processes in glioblastomas can be visualized with different advanced MR techniques. Combining maps of oxygen metabolism (CMRO_2_), relative cerebral blood volume (rCBV), vessel size imaging (VSI), and apparent diffusion coefficient (ADC) or amide proton transfer (APT) can provide early information on tumour infiltration and high-risk regions of future recurrence. Oxygen consumption is increased 6 months prior to tumour progression being visible on conventional MR imaging. However, presence of the Warburg effect, marking a switch from an infiltrative to a proliferative phenotype, could result in CMRO_2_ to appear unaltered in high-risk regions. Including information on biomarkers representing angiogenesis (rCBV and VSI) and hypercellularity (ADC) or protein concentration (APT) can omit misinterpretation due to the Warburg effect. Future research should evaluate these biomarkers in radiotherapy planning to explore the potential of advanced MR techniques to personalize target volume delineation with the aim to improve local tumour control and/or reduce radiation-induced toxicity.

## Introduction

Patients diagnosed with glioblastoma face poor prognosis, as the median survival from initial diagnosis is less than 15 months [1]. Current standard practice in management of glioblastoma comprises maximum safe tumour resection or biopsy followed by postoperative chemoradiotherapy and adjuvant chemotherapy [2]. Delineation of the gross tumour volume (GTV) and the clinical target volume (CTV) required for radiotherapy planning is performed on a combination of computed tomography (CT) and magnetic resonance (MR) imaging only visualizing macroscopic features of the tumour [3]. Delineation on CT only has shown insufficient tumour coverage and can miss 20% of the target volume delineated on MR imaging [4]. Therefore, (contrast-enhanced) T1-weighted and T2-weighted/FLAIR sequences at 1.5 or 3 Tesla (T) are fused with the planning CT for radiotherapy planning. Microscopic tumour infiltration, which is a key characteristic of glioblastomas, however, cannot be fully visualized by these structural MR sequences. Hence, in current clinical practice, an expansion of 10–20 mm is applied to the GTV in every direction to cover for nonvisible tumour invasion to define the CTV [5, 6]. Ultra-high field MR imaging at field strengths of 7 T or higher is promising for visualization of glioblastoma infiltration and thus improving target volume delineation, but is not yet regularly available in clinical practice [7, 8]. Using 7 T systems for neuro applications has recently gained FDA and CE approval, which is an important step towards future routine clinical use of ultra-high field MR imaging for glioblastoma imaging diagnostics. However, in the remained of this review, the main focus will be on literature considering advances on the current clinical standard using 1.5 and 3 T systems.

An opportunity arises with advanced MR techniques, which offer the ability to visualize pathophysiological properties of tumours. These physiological processes might precede morphological changes on conventional MR imaging and thus provide information on microscopic tumour infiltration and/or high-risk regions for future relapse. With this information, the 10–20 mm isotropic expansion would be unnecessary as the CTV can be delineated, so that only the infiltration and high-risk regions are included. An improved CTV delineation could eventually lead to improved local tumour control and/or reduced radiation toxicity.

In this review, the aim is to evaluate promising advanced MR techniques that have potential to improve CTV delineation for radiotherapy of glioblastoma in clinical practice. Key pathophysiological processes in glioblastoma development are discussed and, thereafter, promising advanced MR techniques are discussed that may visualize these physiological processes.

## Understanding glioblastoma development

Progression of glioblastoma is associated with hypoxic tumour microenvironment that is known to exist within glioblastomas. At cellular level, the glioblastoma stem-like cells (GSC), a specific subpopulation of cells that display principal stem cell properties like self-renewal and differentiation, thrive in harsh microenvironmental niches [9–11]. Hypoxia and hypoxia-inducible factors (HIFs) play a critical role in creating the microenvironment that promotes cellular interactions and signaling pathways required for the survival and self-renewal of GSCs [12]. As GSCs are highly resistant to radiotherapy and chemotherapy, it is believed that survival of GSCs play a major role in the development of recurrence after treatment [13–15].

**Fig. 1:**
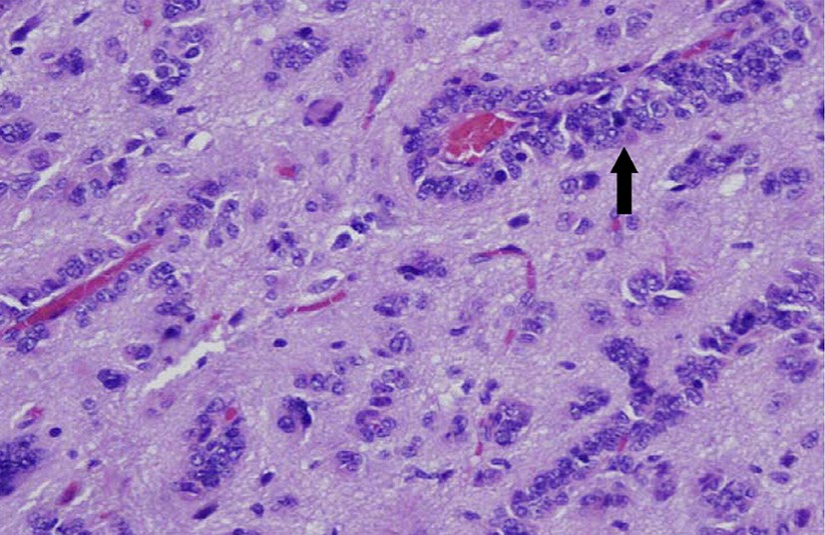
Vessel co-option (black arrow) involves organization of tumour cells into cuffs around normal microvessels (adapted with permission from Hardee et al*.* [118])

Hypoxia is commonly observed in solid tumours, being a natural consequence of increased oxygen diffusion distance due to tumour expansion. In the early 70s, Folkman et al. first proposed that angiogenesis is vital for the progression of solid tumours beyond a size of a few mm^3^, as tumour expansion demands an increase in supply of oxygen and nutrients [16]. Emerging evidence, however, shows that another mechanism called vessel co-option can act as an alternative for tumour blood supply [17]. Vessel co-option is a process in which pre-existing vasculature is hijacked by tumour cells that form cuffs around microvessels. In a preclinical study, Holash et al*.* first demonstrated vessel co-option in gliomas to precede angiogenesis by up to 4 weeks (see Fig. 1) [18]. In this study, vessel co-option was shown to be associated with an upregulation of angiopoietin-2 (Ang-2), a growth factor that belongs to one of the main pathways involved in angiogenesis. The Ang-2 overexpression was associated with vascular endothelial cell apoptosis and vessel regression, which occurs in Ang-2 overexpression in the absence of vascular endothelial growth factor (VEGF) [19–21]. Subsequently, this led to a temporary avascular tumour with increasing hypoxia, which in turn promotes induction of VEGF, a growth factor that promotes migration and proliferation of endothelial cells and stimulates sprouting of new blood vessels. This marks a switch from vessel co-option as the preferred mechanism of vascularization to angiogenesis. The process of angiogenesis will remain dominant due to the continuous presence of hypoxia, and eventually lead to an abnormal vascular network with dilated vessels, abnormal perfusion, and excessive leakiness. In addition to angiogenesis, it is believed that the hypoxic switch promotes another mechanism for neovascularization: vasculogenesis [22]. This process involves the mobilization, differentiation, and recruitment of circulating bone marrow-derived cells for the de novo formation of tumour vasculature.

Glioblastoma cells can adopt and switch between an infiltrative phenotype, where cells migrate in a saltatory fashion, and a proliferative phenotype resulting in local tumour growth [23]. When a proliferative phenotype is adopted, altered metabolism in glioblastomas can result in rapid cell proliferation. In normal differentiated cells, glucose is metabolized into pyruvate, which subsequently enters the tricarboxylic acid cycle to generate adenosine triphosphate (ATP) through a process called oxidative phosphorylation. ATP production is required for ATP hydrolysis, in which crucial energy is released for numerous cellular processes. Oxidative phosphorylation maximizes ATP production (36 mol ATP / mol glucose), with minimal production of lactate. However, when oxygen is limited, oxidative phosphorylation cannot occur and anaerobic glycolysis results in inefficient ATP production (2 mol ATP/ mol glucose) and the production of large amounts of lactate [24]. Warburg et al. observed that malignant cells tend to convert a majority of glucose to lactate, regardless of the presence of oxygen, resulting in less-efficient ATP production (4 mol ATP/mol glucose) when compared to oxidative phosphorylation [25]. This process is called aerobic glycolysis or the Warburg effect and is also observed in glioblastomas [26]. Although it is hypothesized that aerobic glycolysis can be beneficial for rapid cell proliferation due to the creation of additional cellular components (e.g., nucleotides, amino acids, and lipids) along with the production of ATP, a conclusive explanation for aerobic glycolysis in cancer cells has remained elusive [24].

## Vascular leakiness

**Fig. 2:**
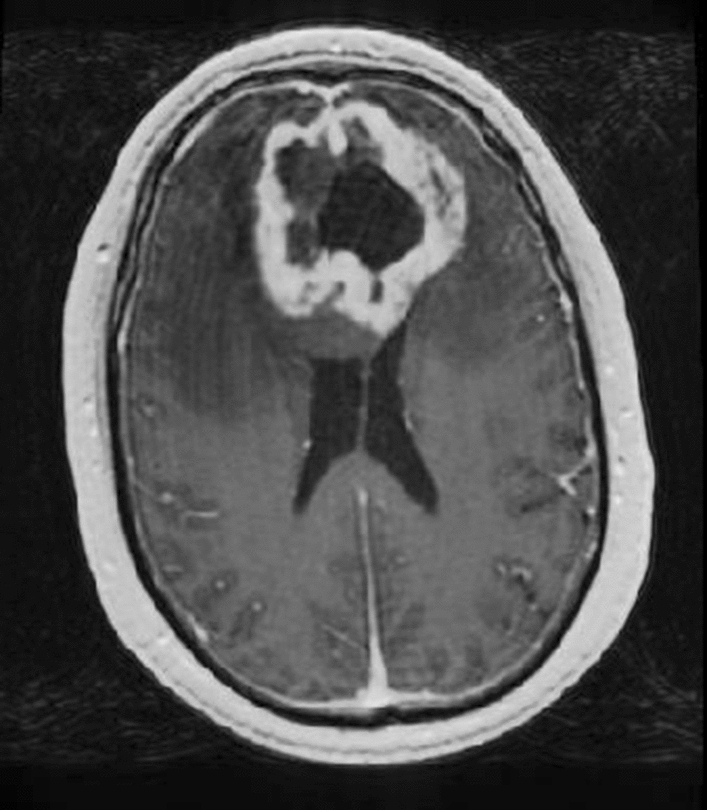
Post-gadolinium-based contrast-enhanced T1-weighted axial image shows a ring enhancing lesion, in this case a glioblastoma. The peripheral enhancement is caused by accumulation of the contrast agent due to disruption of the BBB

In the healthy brain, the blood–brain barrier (BBB) usually restricts exogenous contrast agents to the vascular bed. These agents are commonly based on low-molecular-weight gadolinium and locally shorten the T1 relaxation times allowing for better contrast between regions with and without the contrast agent. Disruption of the BBB, e.g., caused by glioblastoma, can lead to accumulation of these agents in the interstitial spaces surrounding the leaky vasculature [27]. As shown in Fig. 2, accumulation can be seen as an increase in signal intensity on T1-weighted MR imaging. Contrast enhancement on this conventional MR image is currently used as a surrogate measure of malignancy and can be indicative of high-grade gliomas [28, 29]. In various studies, progression-free survival and overall survival for both low-grade and high-grade glioma patients were observed to be worse when contrast enhancement was present [30–34]. In current clinical practice, enhancing areas on gadolinium-based contrast-enhanced T1-weighted MR imaging are by default included in the GTV delineation of glioblastoma patients [3].

### Dynamic contrast-enhanced MR imaging

Additional information on the presence of BBB disruption can be gained with dynamic contrast-enhanced (DCE) MR imaging. The principles of DCE-MR imaging are based on the exchange of gadolinium-based contrast agents between the intravascular compartment and the interstitial tissue. By measuring the time course of the contrast agent as it diffuses from the blood pool into tissue, information on flow and permeability of the vasculature can be obtained. DCE-MR imaging can be used to study the degree of vascular leakiness and has great potential to monitor changes in vascular permeability arising from antiangiogenic therapies [35].

Various DCE kinetic parameters in regions of tumour infiltration are shown to correlate to microvascular parameters that are involved in disruption of the BBB. In a biopsy study, Keil et al. explored the potential of DCE-MR imaging to predict vascular growth factor expression and neovascularization in 30 glioma patients [36]. In regions with vital tumour, tumour infiltration, and healthy brain tissue, 120 biopsies were taken to evaluate the parameters involved in BBB disruption with DCE kinetic parameters (i.e., the contrast agent efflux transfer constant, the contrast agent reflux transfer constant, the extracellular–extravascular volume fraction, and the intravascular volume fraction). Significant correlations were found not only in vital tumour tissue, but also, in a lesser degree, in the histologically confirmed infiltration zone highlighting the potential of DCE-MR imaging to visualize tumour infiltration.

Pattern of failure was evaluated in 52 glioblastoma patients who underwent both diffusion-weighted imaging (DWI) and DCE-MR imaging by Wahl et al. [37]. Progression during follow-up was seen in 33 patients and delineated as the recurrence volume to assess the overlap with regions with increased perfusion and/or restricted diffusion. The median proportion of the recurrence volume within the region with elevated cerebral blood volume (CBV) was 22%, when combined with restricted diffusion the median proportion increased to 30%. Based on these results, the authors implied that the majority of failures occur in regions characterized by neither restricted diffusion nor increased perfusion. The median proportion of the overlapping volume (i.e., both restricted diffusion and increased perfusion) that developed progression during follow-up was 77%, indicating that regions with restricted diffusion and increased perfusion are likely to show progression in the future. This overlapping volume has been targeted in a phase II dose escalation study that showed significantly improved 12-month overall survival (92%) compared to historical control (*p* = 0.03) [38].

## Angiogenesis

Malignant gliomas are characterized by a high degree of angiogenesis, resulting in increased neovasculature and vasodilatation [39]. Histologically, the abnormal number of tumour vessels can be measured by determination of the microvascular density [40]. This process, however, requires tissue sampling and can be time-consuming [41]. Advanced MR imaging offers an alternative to histological assessment of the microvasculature; dynamic susceptibility contrast (DSC) MR imaging and arterial spin labelling (ASL) are two perfusion MR techniques that can provide valuable information on the degree of angiogenesis within the brain.

### Dynamic susceptibility contrast MR imaging

DSC-MR imaging exploits the susceptibility-induced signal loss that is caused by paramagnetic contrast agents like gadolinium-based compounds. Rapid repeated imaging of the brain is performed after intravenous injection of a bolus of contrast agent; attenuation of the signal intensity caused by the susceptibility provides information on the amount of contrast in the microvasculature. This information can be used to calculate various perfusion parameters. Within gliomas, the relative cerebral blood volume (rCBV) has been shown to correlate significantly with microvascular density [42].

The positive correlation of the degree of tumour infiltration in gliomas and rCBV is shown to predict tumour infiltration with a higher accuracy than various biomarkers acquired with MR Spectroscopy (MRS). In an image-guided biopsy study with 13 patients, Hu et al. demonstrated the potential of rCBV to distinguish tumour progression in high-grade gliomas (sensitivity = 91.7% and specificity = 100%) [43]. This finding was supported by Price et al., who presented a significant correlation between mean rCBV and the tumour proliferation index (MIB-1) in 10 patients with high-grade gliomas (*r* = 0.66, *p* < 0.001) [44]. In four patients where biopsies went outside the region of contrast enhancement, the mean rCBV at the biopsy site was increased: in three patients, the increased rCBV extended 1 cm from the edge of enhancement, and in the other patient, the biopsy was taken 2 cm from the enhanced area. In all four patients, the histology of these areas revealed regions of microscopic tumour invasion into normal appearing brain tissue. In subsequent research by the same research group, a comparison of rCBV with various MRS biomarkers as predictor of tumour infiltration in glioblastomas was made (*n* = 50) [45]. With a sensitivity and specificity of 82%, rCBV could predict tumour infiltration with better accuracy than all examined metabolite biomarkers.

**Fig. 3:**
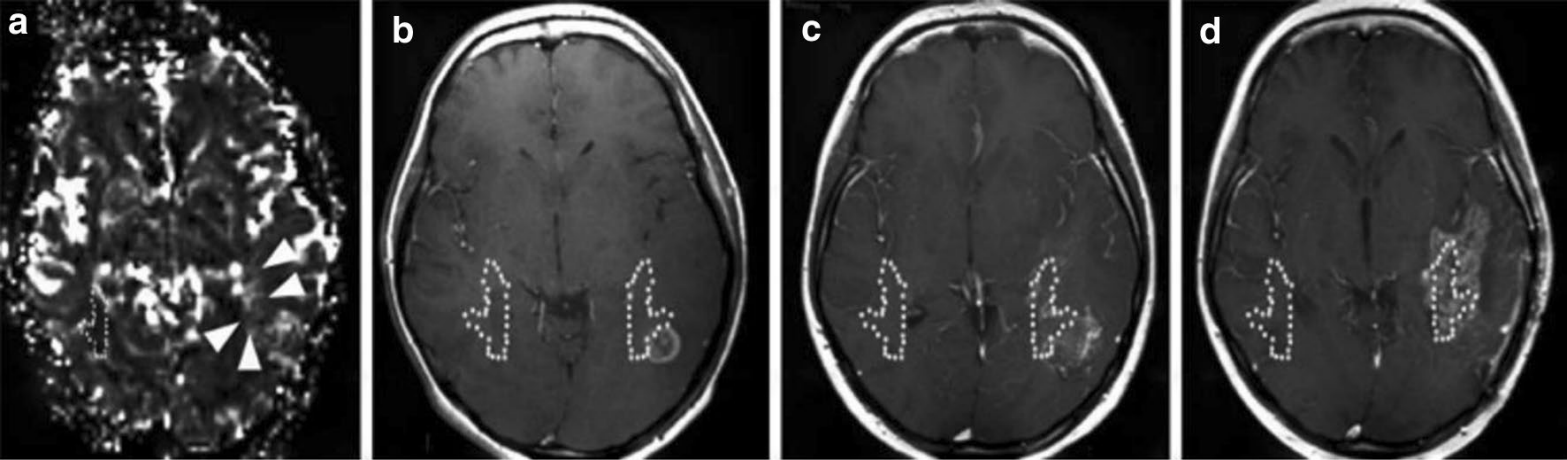
On rCBV map: **a** a specific directional stripe-like pattern that extended beyond the contrast-enhancing tumour rim, was visible (white arrows). Contrast-enhanced T1-weighted imaging of the baseline MR scan (**b**), and the 3 (**c**) and the 6 months (**d**) follow-up MR scan show that this extension (yellow delineation) was covered with contrast enhancement in its entirety after 6 months. The delineation on the contralateral side marks a contralateral reference (reprinted with permission from Blasel et al*.* [48])

In addition to the correlation with tumour infiltration, various studies have shown that increased rCBV can be an indication for high-risk regions of future relapse. Stadlbauer et al. demonstrated changes in rCBV at the site of recurrence 120 days prior to radiological recurrence [46]. From that time-point, a continuous increase was seen in rCBV before indications of progression on structural MR imaging could be seen. The feasibility of rCBV as predictive biomarker for progression was also supported by Stecco et al., where both diffusion tensor imaging (DTI) parameters [apparent diffusion coefficient (ADC) and fractional anisotropy (FA)] and perfusion (rCBV) were investigated in 17 patients [47]. Compared to the contralateral normal appearing white matter (NAWM), significantly higher rCBV values were seen on pretreatment imaging at the site where recurrence occurred during follow-up (*p* < 0.001). This was the case not only for regions that showed enhancement on contrast-enhanced T1-weighted imaging before treatment, but also for regions that did not show enhancement. In addition, a specific directional stripe-like pattern of rCBV increase in a region adjacent to contrast enhancement was investigated by Blasel et al. [48]. In this study, this extended increase of rCBV in a direction away from the tumour border was named the striate sign and observed in 42% of the cases between 2008 and 2009 at their institution (31 out of 77 histologically proven glioblastomas); see Fig. 3. Sixteen patients with tumour recurrence were investigated retrospectively in this study. Four patients underwent second surgery where biopsy of the area of the striate sign revealed glioblastoma cell infiltration in all cases. The entire area of the striate sign showed contrast enhancement, indicating progression, in 15 out of 16 patients after 9 months. This study shows that the striate sign may depict future tumour progression and can extend far beyond the margins of contrast-enhancing tumour.

When performing DSC-MR imaging with a gradient and spin echo acquisition, the acquired images can also be used to perform vessel size imaging (VSI) [49]. DSC-MR imaging can thus provide information on increased microvascular density and vasodilatation and therefore is a promising tool to assess the degree of angiogenesis within glioblastomas.

**Fig. 4:**
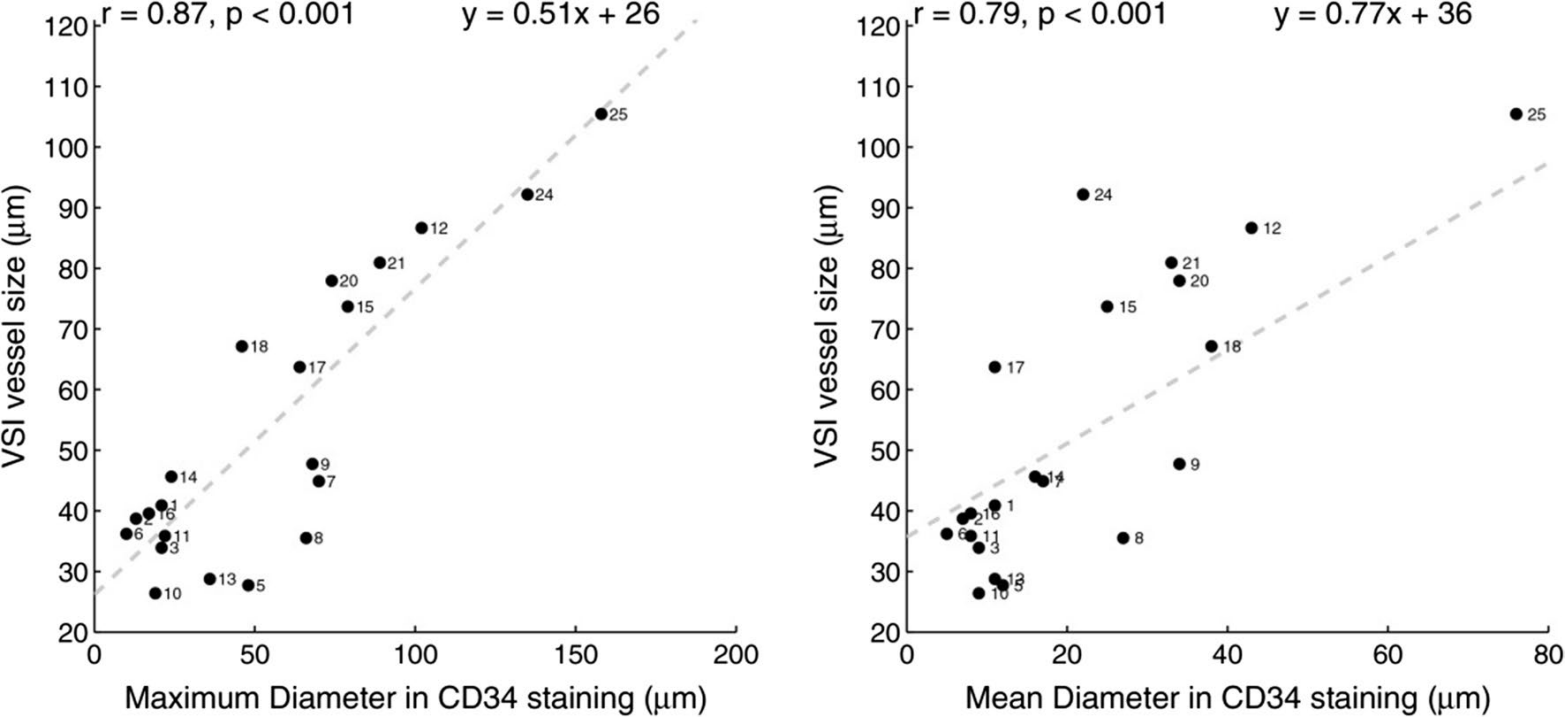
Median vessel size estimated with VSI compared with the diameter obtained from CD34 staining shows a positive correlation for both the maximum (left) and the mean diameter (right). Although both significant, the maximum diameter shows a better correlation (reprinted with permission from Kellner et al*.* [50])

In a prospective stereotaxic biopsy study by Kellner et al., the vessel size index acquired with VSI was proven to be related to histologically increased vessel diameters in human gliomas [50]. Although an overestimation of normal vessel size and an underestimation of grossly enlarged vessels were observed, a significant correlation for both mean and maximum vessel size with histologically measured diameters was seen (see Fig. 4). Chakhoyan et al. corroborated these findings, concluding that MR-based vessel size measures accurately reflect vessel caliber within high-grade gliomas, while the traditional measures of rCBV are correlated with vessel density rather than vessel size [49].

An increase in vessel size can be an indication of high-risk sites of future recurrence. Vessel size imaging was acquired by Stadlbauer et al. during the follow-up of 56 patients who received standard treatment for glioblastoma [46]. Approximately 120 days prior to radiological recurrence, a decreasing vessel size index was observed for a duration of 80 days at the site of relapse. After this decrease, 40 days before radiological recurrence, the vessel size index was shown to continuously increase. As a continuous increase in mean vessel density was seen 120 days prior to radiological recurrence, the authors hypothesized that early angiogenic activity was dominated by the formation of smaller vessels, which transformed into larger lumen vessels during later phases of tumour vascular development.

### Arterial spin labelling

**Fig. 5:**
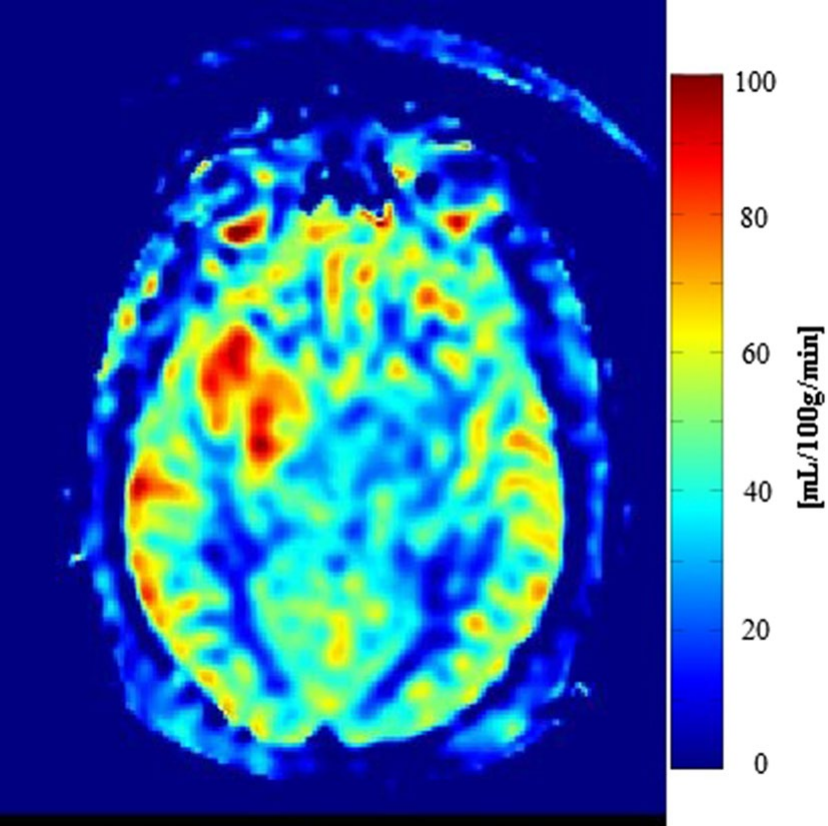
ASL-CBF shows increased perfusion in a patient with a glioblastoma in the right frontotemporal lobe

An alternative parameter to assess vascular proliferation within glioblastoma is regional cerebral blood flow (CBF). Relative CBF has shown a positive correlation with rCBV and microvascular density as an increase in CBF can be observed in high-grade gliomas; see Fig. 5 [51, 52]. Arterial spin labelling (ASL) is a non-invasive MR technique used to measure CBF and was found useful for non-invasive glioma grading [53]. By magnetically labelling the water molecules in arterial blood, which then flows into the brain, a tagged image of the brain can be acquired. Subtraction between labeled and control images creates images with signal weighted by cerebral perfusion that via a kinetic model can be quantified to generate images of CBF [54].

In a biopsy study, ASL guidance was found to improve the accuracy of target selection for stereotaxic biopsies when combined with MRS [55]. A conclusive diagnosis could be made for 28 out of 30 patients (93.3%).

### Tumour metabolism

Like in most malignancies, glioblastomas rewire cell metabolism to stimulate excessive growth and to ensure survival [26]. With various advanced MR techniques, information on different aspects of tumour metabolism can be gained. Altered oxygen consumption can be assessed using quantitative blood-oxygen-level-dependent (qBOLD) MR imaging. A promising technique to visualize elevated protein concentrations in tumour regions is amide chemical exchange saturation transfer (CEST) imaging, also known as amide proton transfer (APT) CEST imaging. Dynamic glucose-enhanced (DGE) MR imaging is a promising technique to assess glucose uptake. Furthermore, the proportion of numerous metabolites can be evaluated with MRS.

### Quantitative blood-oxygen-level-dependent MR imaging

Angiogenesis in glioblastoma is characterized by dysfunctional microvascular proliferation resulting in hypoxic foci [56]. Subsequently, the hypoxic environment results in an altered cerebral metabolic rate of oxygen (CMRO_2_), which is particularly observed in high-grade tumours compared to normal brain tissue. Oxygen-17 MR imaging is a technique where the patient inhales Oxygen-17 in enriched form, allowing direct measurement of the CMRO_2_ in glioblastoma patients at 7 T [57, 58]. Due to the relatively low signal sensitivity, however, MR acquisition of glioma patients at clinical field strengths is yet to be investigated. Functional MR imaging, on the other hand, relies on the magnetic susceptibility of blood that depends on the oxygenation state of hemoglobin (Hb) [59]. qBOLD MR imaging is a functional MR method that is used to measure the oxygen extraction fraction (OEF) from the complex relationship between T2* and the deoxygenated Hb [60]. Subsequently, combining information on the OEF with CBF allows for indirect mapping of the oxygen consumption, CMRO_2_ [61].

Altered oxygen consumption can identify regions that will form relapse up to 6 months later in glioblastoma patients. Kim et al. included ten healthy subjects and ten glioblastoma patients to compare cerebral oxygen consumption [62]. A significant increase of 59% in CMRO_2_ was seen in contrast-enhancing tumour compared to healthy tissue. Peritumoural tissue showed an increased oxygen consumption of 27%. These findings were supported by Stadlbauer et al., who observed an increased oxygen consumption in high-grade gliomas compared to both the contralateral and ipsilateral NAWM (*p* < 0.001) in 82 glioma patients [63]. The same research group evaluated advanced MR follow-up examinations of 56 glioblastoma patients who underwent standard treatment [46]. They found that CMRO_2_ started to increase 190 days prior to relapse at the site of recurrence. At 60 days before relapse, a maximum was reached; thereafter, the CMRO_2_ was observed to decrease. The tissue oxygen tension, which is influenced by CMRO_2_ amongst others, started to decrease (i.e., hypoxia) 190 days before relapse, reaching a minimum value at 90 days prior to recurrence. Afterwards, the tissue oxygen tension was observed to increase until recurrence occurred.

### Amide chemical exchange saturation transfer imaging

**Fig. 6:**
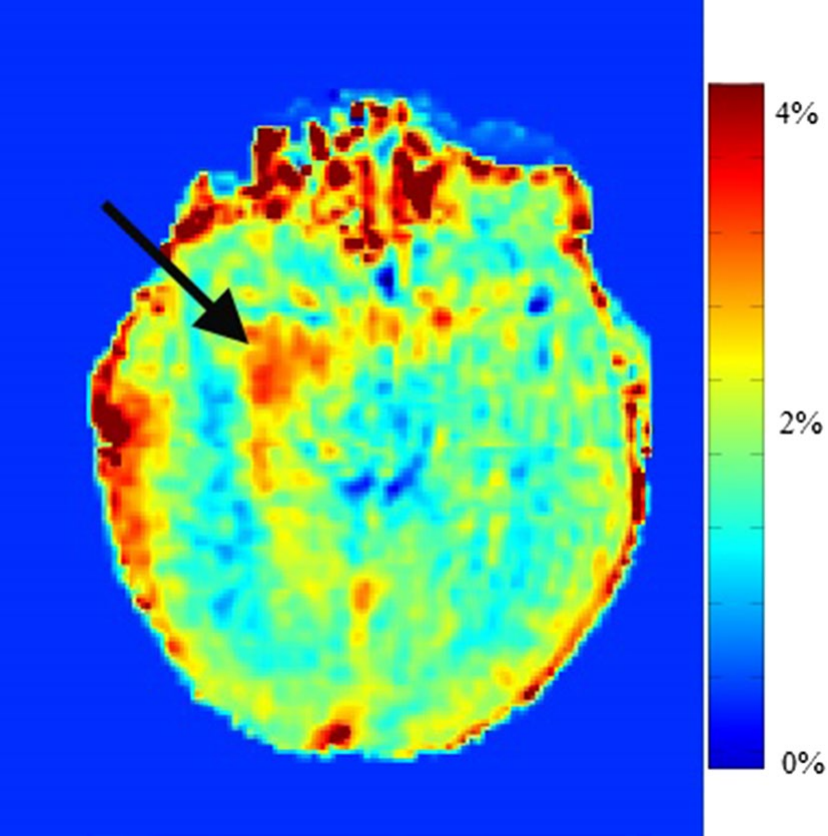
APT imaging shows an increased signal intensity in the tumour region (black arrow). This signal intensity is mainly caused by the increased concentration of proteins in this region

APT CEST imaging can be used to gain information on the presence of amide protons. In CEST, the concept of saturation, i.e., a temporary state in which tissue shows no net magnetization is exploited to achieve image contrast. By applying a radiofrequency pulse at the resonant frequency of a chemical species of interest, this chemical species can be targeted as it will show reduced signal due to the saturation effect. However, most chemical species are present in significant smaller quantities compared to water, meaning that the signal change would be unnoticeable. Various chemical species, in the case of APT imaging amides (–NH), have a proton in its structure that is exchangeable with those of the free water pool. When saturated, the magnetic saturation of the amides will spontaneously be transferred to water over time, due to chemical exchange of the excited amide protons with non-excited water protons. The proton of the amides will thus be replaced with an unsaturated proton from water, which can in turn be saturated for another transfer. By continuously saturating the amides the continuous transfer of excited protons will lead to a buildup of saturation in water. This decrease in water signal can indirectly measure the concentration of amides in a target area [64]. The major known contributors to the APT signal are proteins and peptides [65]. Therefore, APT imaging can provide information on increased local protein concentration, which may be a result of sustained cell proliferation (one of the hallmarks of cancer) and increased cell density [66]. In gliomas, APT signal intensity was shown to be a promising non-invasive tool to predict the presence of isocitrate dehydrogenase (IDH) mutation, which is associated with favourable prognosis [67, 68]. For prediction of the IDH mutation status in low-grade gliomas, an area under the receiver-operator characteristic curve (AUC) of 0.89 was observed when evaluating the maximum APT signal intensities in multiple regions of interest [69]. In a prospective study, Paech et al. acquired CEST MR imaging of 31 glioma patients at 7 T and showed that APT CEST imaging at 7 T has the ability to predict IDH mutation status in both low-grade and high-grade gliomas (AUC = 0.98) [70]. 7 T APT signal intensity was also observed to be associated with overall survival and progression-free survival in 26 newly diagnosed high-grade glioma patients, highlighting the potential of APT imaging to investigate the prognostic value [71]. An example of APT imaging is shown in Fig. 6.

APT CEST can be used to distinguish true glioma tissue from healthy tissue or post-treatment effects. Preclinical research by Zhou et al. first explored the potential of APT CEST to distinguish viable glioblastoma from post-treatment radiation necrosis [72]. Histologically confirmed regions with malignant tissue in 9 rats showed significantly higher APT signal intensity than regions with radiation necrosis or contralateral NAWM (both *p* < 0.001). Sagiyama et al. observed a significantly lower APT signal intensity in a group of mice treated with TMZ (*n* = 6) compared to a control group (*n* = 5) [73]. Histological evaluation determined no differences in cell density or apoptosis rate; however, a significantly lower Ki-67 labelling index was seen in the treated group (*p* < 0.0001). The correlation between APT signal intensity and the Ki-67 labelling index was first confirmed in human gliomas by Togao et al. [74]. In 36 glioma patients, APT imaging was performed at 3 T to compare APT signal intensities with tumour grade. The mean APT signal intensities were 2.1 ± 0.4% for grade II gliomas, 3.2 ± 0.9% for grade III gliomas, and 4.1 ± 1.0% for glioblastomas. APT signal intensity was positively correlated with Ki-67 labelling index (*r* = 0.43, *p* < 0.01) and cell density (*r* = 0.38, *p* < 0.05). These correlations were confirmed by Jiang et al. [75].

These biopsy studies and the ability of APT CEST to distinguish tumour tissue from treatment effects can be used to predict areas at risk for future tumour progression. Mehrabian et al. acquired APT CEST imaging at 3 T before, during, and after chemoradiation therapy in 19 glioblastoma patients [76]. They found that APT CEST could predict future tumour progression as early as 2 weeks into treatment. The utility of APT CEST acquisition at 1.5 T was examined in a prospective study (*n* = 51) by Chan et al., who found significant differences in quantitative CEST parameters in patients that developed tumour progression within 6.9 months compared to those with late tumour progression [77]. These findings were supported by Regnery et al., who found APT CEST imaging to yield significant predictors of early progression at 7 T [78].

Another effect that can be assessed with CEST imaging is nuclear Overhauser enhancement (NOE), an effect dominated by the presence of exchangeable protons in aliphatic groups in macromolecules with resonance frequencies shifted upfield from water, i.e., ranging from − 2 to − 3.5 ppm. Although often NOE is seen as contaminating APT CEST imaging, in particular when magnetization transfer asymmetry ratios are used to assess APT signal for which radiofrequency pulses with both + 3.5 and − 3.5 ppm off resonance frequencies are required, using CEST acquisition and analysis techniques that separate NOE signal, are gaining momentum in glioblastoma imaging. Preliminary work illustrated in three patients with newly diagnosed glioblastoma that NOE CEST was correlated to histopathological assessment of cell density [79]. In line with this finding and in similar fashion as APT CEST imaging, it has been shown that NOE CEST is linked to prognosis [70, 78] and grading [80] of glioma/glioblastoma and, potentially, can serve as an early biomarker (i.e., 2 weeks into treatment) of tumour progression as well [76, 78]. Although the latter study did not report on differences in NOE CEST between true progression and pseudo-progression, this has been reported in patients being treated for brain metastases [81].

### Dynamic glucose-enhanced MR imaging

Besides gadolinium-based contrast agents, which are the most commonly used contrast agents in neuro-oncological MR imaging, alternative contrast agents have been increasingly investigated. DGE-MR imaging uses d-glucose as a biodegradable contrast agent and has the aim to visualize glucose uptake or glucose metabolism [82]. Paech et al. acquired DGE-MR imaging at 7 T from nine glioblastoma patients; when comparing the glucose-enhanced tumour region with contralateral NAWM, the median signal intensity was observed to be significantly higher (2.02% vs 0.08%, *p* < 0.0001) [83]. Clinical application of DGE-MR imaging may be challenging, as acquisition at clinical field strengths (e.g. 3 T) was shown to yield smaller effect sizes and can be more sensitive to motion artefacts [84, 85].

### MR spectroscopy

Metabolites, the intermediate or end products of metabolism, play a crucial role in cell growth, development, and reproduction. Reprogramming of cellular metabolism is considered one of the emerging hallmarks of cancer [66]. Conventional MR imaging relies on free protons, which are most abundant in water, to generate its signal. The signal, however, is also affected in a much lesser degree by protons bound to macromolecules. These bound protons have specific frequency variations. After suppression of the water signal, MRS can acquire data on separated frequency peaks, each representing a specific macromolecular component. Assessment of the peaks and ratios from various metabolites can provide information on a wide range of metabolic processes, such as energy metabolism, cell proliferation, and necrotic tissue changes. Commonly used metabolites for proton MRS are *N*-acetyl aspartate (NAA), choline (Cho), creatine/phosphocreatine (Cr), lactate (Lac), and lipids (Lip) [86, 87]. As metabolic changes may precede anatomic changes, proton MRS is a promising technique to determine early tumour development [88]. Within high-grade gliomas, increased Cho and/or decreased NAA is commonly observed, making these metabolites promising for visualization of glioblastoma infiltration [89].

Histologic evaluation of glioma tissue has shown the capability of proton MRS to distinguish tumour infiltration. Croteau et al. investigated the correlation of proton MRS metabolic ratios and the degree of tumour infiltration in 31 untreated glioma patients [90]. In 247 tissue samples, a significant correlation of Cho/nCho (*p* = 0.0372) and Cho/NAA (*p* = 0.0018) was found with MIB-1, a cellular proliferation index indicating tumour infiltration. Matsumura et al. evaluated the correlation between Cho concentration and MIB-1 in 14 glioma patients with single-voxel proton MRS [91]. Although Cho concentration is believed to be elevated in glioma tissue, they only found a significant correlation for low-grade gliomas. This might be caused by the heterogeneous nature of high-grade gliomas, particularly for glioblastomas. Due to the large voxel size in single-voxel proton MRS, it is almost unavoidable to exclude all necrotic tissue during the selection of a voxel of interest.

**Fig. 7:**
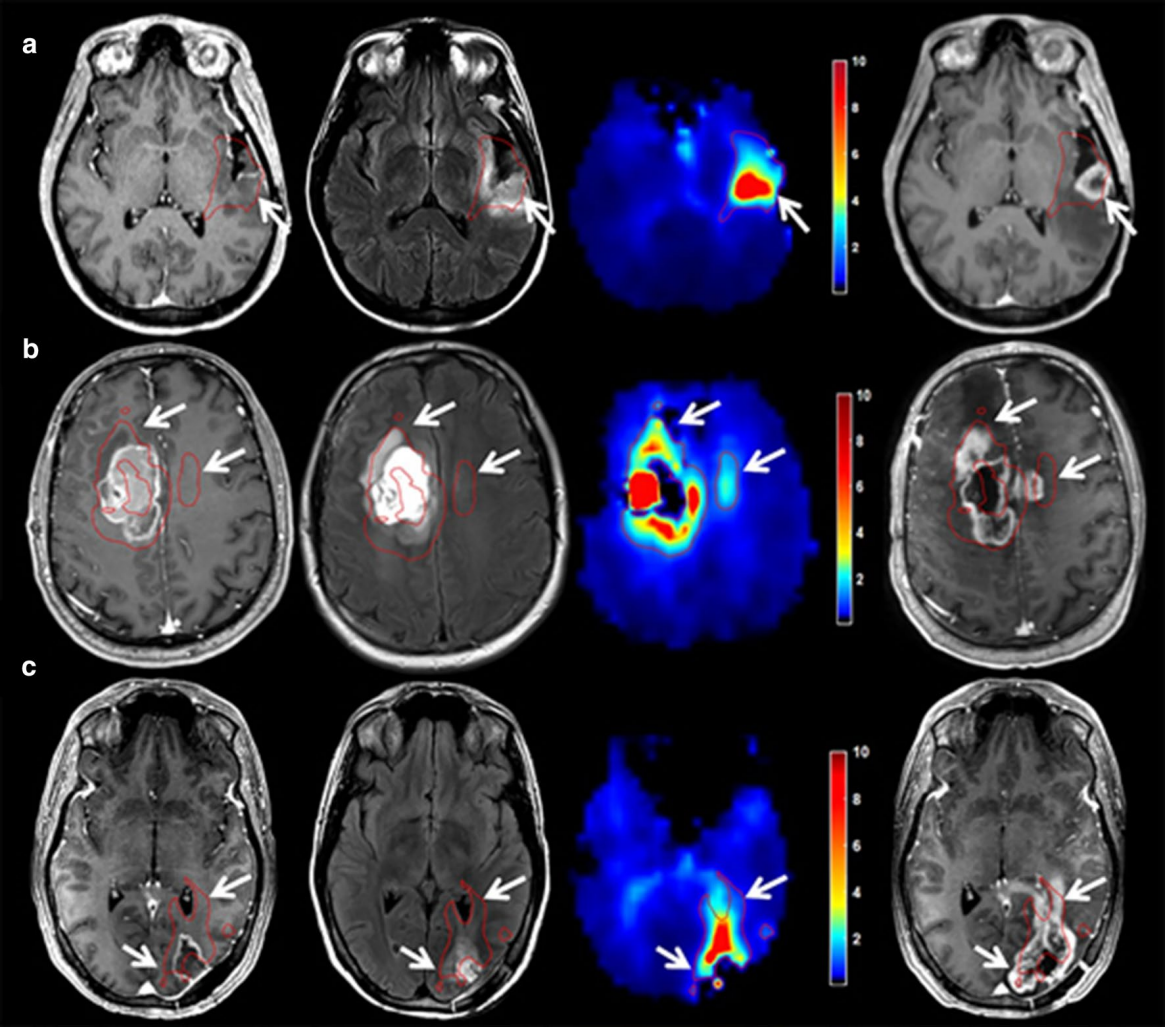
Pre-radiotherapy Cho/NAA abnormalities were observed and delineated (red contour). Pre-RT imaging shows that increased Cho/NAA ratios on the Cho/NAA map (third column) extended beyond the GTV on T1-weighted contrast enhancement (first column) and T2-weighted/FLAIR (second column). First recurrence on contrast-enhanced T1-weighted imaging after radiotherapy (fourth column) showed contrast-enhancing tumour development within the pre-radiotherapy increased Cho/NAA volume in 3 patients (**a**–**c**) (reprinted with permission from Cordova et al*.* [93])

Multi-voxel MRS has the potential to identify and delineate substantial tumour infiltration and high-risk regions for recurrence. The feasibility of target delineation with multi-voxel proton MRS was first explored by Pirzkall et al. [92]. On average, the volume delineated on proton MRS was 58% of the volume based on T2-weighted imaging. Metabolic active disease (i.e., regions with abnormal Cho/NAA ratios), however, was still observed to extent ipsilaterally beyond the T2-weighted delineation in 9 out of 12 patients with glioblastoma. On contrast-enhanced T1-weighted MR imaging, the extension beyond the T1-weighted volume was not as great as was seen with grade III patients. Cordova et al. developed an imaging pipeline utilizing high resolution (0.1 cm^3^ nominal voxel size) spectroscopic imaging to generate whole-brain metabolic maps and evaluated the correlation between proton MRS biomarkers and Sox2 density, a normalized metric of tumour infiltration, in tissue samples of 13 patients with glioblastoma [93]. Various metabolic markers showed significant correlations, but Cho/NAA exhibited the strongest association with tumour infiltration (*ρ* = 0.82, *p* < 0.0001). During follow-up of these patients, 5 of the 13 patients had T1-weighted contrast-enhancing progression at the time of analysis. All patients showed T1-weighted contrast-enhancing progression in regions that exhibited Cho/NAA abnormalities before radiotherapy was given (see Fig. 7). A similar correlation between regions with increased Cho/NAA and recurrence site was also observed by Park et al. and Czernicki et al*.* [94, 95].

Besides proton MRS, phosphorous MRS could also be a promising technique. This technique can be used to detect metabolites that play a key role in energy metabolism and, additionally, was shown to enable non-invasive pH measurements of the human brain at 7 T [96]. Also with clinical field strengths, phosphorous MRS can provide valuable information. Walchhofer et al. observed regional differences of energy metabolism in 32 patients with newly developed glioblastoma at a field strength of 3 T [97]. Additionally, Wenger et al. found that, in patients with recurrent glioblastoma treated with bevacizumab, the intracellular pH was significantly higher in regions that were to progress compared to control areas (*p* < 0.001) [98].

## Hypercellularity

High-grade gliomas show increased cellular density, which impedes free water diffusion [99]. Diffusion-weighted imaging (DWI) is an MR technique where free water molecular diffusion is measured; therefore, DWI is a promising tool to visualize glioblastoma infiltration.

### Diffusion-weighted imaging

**Fig. 8:**
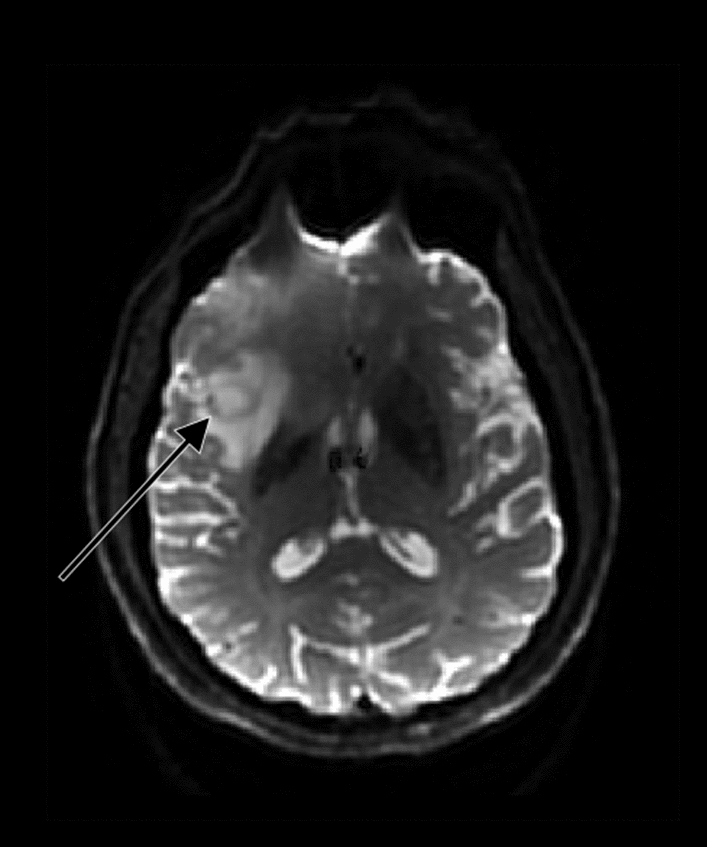
Glioblastomas show decreased ADC values (black arrow) on the ADC map due to increased cell density. In the center of the tumour, the ADC values are higher. This is explained by the necrotic center that is characteristic for glioblastomas

During DWI, gradient pulses are applied in such way that water molecules that do not move between pulse applications are refocused and generate signal. Moving water molecules along the direction of the applied gradient pulses will cause dephasing, which results in hypointense DWI signal. On DWI areas of restricted diffusion, therefore, appear bright, while areas of free water motion appear dark. By varying the gradient strengths, water molecules that diffuse with different speeds can be measured to create images with varying diffusion weighting. These resulting images can be used to calculate an ADC, with lower ADC values reflecting lower diffusion. Various pathophysiological properties of high-grade gliomas can influence ADC values. Necrosis, one of the characteristics of glioblastomas, shows higher ADC values as there is more free diffusion of water molecules. In solid tumour tissue, the size of the extracellular space is limited by the increased cell density, lowering diffusion and thus ADC values (see Fig. 8) [100]. Therefore, DWI has the potential to be used as an indirect measurement of abnormal cellularity in glioblastomas.

Biopsy studies have shown that DWI is not helpful for the distinction between glioma infiltration and peritumoural brain tissue. The correlation between tumour cellularity and ADC value, which can be derived from DWI, in solid tumour tissue has been histologically proven by Kono et al. [101]. The ADC in solid tumour tissue was found to be capable of predicting tumour grade in gliomas; the ADC was significantly higher in low-grade astrocytomas when compared to glioblastomas (1.14 vs 0.82). Signal intensities on DWI and ADC maps in peritumoural hyperintense areas on T2-weighted imaging, however, were not useful in determining the presence of peritumoural neoplastic cell infiltration. These findings were supported by Pauleit et al., who concluded that distinction of tumour infiltration and adjacent edema or reactive gliosis in peritumoural tissue in individual patients with gliomas cannot be done using DWI [102].

Analysis of the recurrence site revealed that increased cell density can identify the site of recurrence at least 3 months before morphologic changes are visible on conventional MR imaging. Gupta et al. evaluated the correlation between low ADC values during treatment and recurrence location during follow-up [103]. Only in 67 of the 208 glioblastoma patients (32.2%), visibly detectable restricted diffusion was seen during treatment. 27 patients showed low ADC lesions and no contrast enhancement. At the site of restricted diffusion, 23 patients (85.2%) developed contrast-enhancing tumour at a median of 3.0 months later. This is congruent with the findings of Elson et al., who observed an overlap of pre-radiotherapy ADC hypointensity volumes with recurrences in 28 out of 32 (88%) cases [104]. The median progression-free survival in patients with ADC hypointensity was 3.2 months. Pramanik et al. showed that the use of pre-radiotherapy ADC maps to predict the location of late progression was inferior to the predictability for early progression [105]. For the 5 patients who progressed within 6 months post-radiotherapy, the median percentage of overlap between the pre-radiotherapy ADC hypointensity volume and the recurrent contrast-enhancing GTV was 78%, whereas 10 patients who progressed after 6 months had an overlap of 53%. Wahl et al. investigated the pattern of failure in 52 glioblastoma patients who underwent DWI and DCE-MR imaging [37]. For the 33 patients with radiographic progression during follow-up, the median proportion of the overlapping volume (i.e., the region that displayed both restricted diffusion and increased perfusion) that developed progression during follow-up was 77%. The median proportion of the recurrence volume within the region with restricted diffusion, however, was only 17%. When combined with the region with elevated CBV, the median proportion increased to 30%. Based on these results, the authors imply that regions that display both restricted diffusion and increased perfusion harbor resistant disease that is likely to progress in the future, but most failures will spatially occur in regions characterized by neither restricted diffusion nor increased perfusion. At the same institute, Kim et al. conducted a phase II study in which patients with glioblastoma received dose intensification against regions with restricted diffusion and elevated perfusion resulting in improved overall survival [38]. Chang et al. used computational big-data modeling to evaluate the potential of ADC and FLAIR signal pre-radiotherapy to predict future recurrence [106]. Areas of future recurrence showed a 9.5% decrease in ADC value (*p* < 0.001) and a 9.2% decrease in signal intensity on FLAIR image (*p* < 0.001). Receiver-operating characteristic curves yielded AUC values of 0.566, 0.726, and 0.741 for ADC-only, FLAIR-only, and a combined model, respectively.

## Tumour invasiveness

A key characteristic of glioblastomas is its extensive, diffuse infiltration of tumour cells. This infiltrative behaviour is not fully visible on conventional MR imaging and is one of the reasons why proper management of gliomas is extremely difficult.

### Diffusion tensor imaging

DTI measures direction and magnitude of water diffusion based on data from a greater number (6 or more) of diffusion directions than DWI (3 directions). Water movement within white matter tracts is mainly restricted across the myelin sheaths, meaning that diffusion is more prevalent parallel to myelinated nerve fibers opposed to transverse [107]. With information on the degree and the direction of diffusional anisotropy, insights into white matter tracts can be gained and utilized to provide tractography data. Consequently, DTI offers information that might be useful in predicting invasive growth patterns of glioblastomas along white matter tracts.

In gliomas, regions with tumour infiltration can be identified with high sensitivity on DTI when information on the isotropic and anisotropic components of the diffusion tensor is split. To evaluate the potential of DTI to visualize tumour cell infiltration within peritumoural regions with increased T2-weighted signal intensity, Tropine et al. compared 20 patients with gliomas to 10 patients with meningiomas, in which no tumour infiltration is expected [108]. Comparison of the peritumoural regions showed no significant differences in FA values, suggesting that reliable differentiation between infiltration and vasogenic edema based on DTI was not yet possible. These findings were in concordance with an image-guided biopsy study performed by Price et al. [109]. Preoperative DTI in 20 patients with gliomas were evaluated with the aim to use DTI to distinguish regions with tumour infiltration in NAWM from regions with normal brain tissue. The normalized FA values in this study could not distinguish infiltrated regions from normal brain tissue (*p* = 0.27). Areas beyond tumour enhancement with a normal anisotropic component and an isotropic component greater than 10% compared to contralateral NAWM, however, could identify tumour infiltration with a sensitivity of 98% and specificity of 81%.

Although tumour infiltration can be visualized with high sensitivity, DTI does not seem useful for prediction of areas of future relapse. The integration of DTI for radiotherapy treatment planning of patients with high-grade gliomas was first explored by Jena et al. [110]. DTI-based plans using a 1 cm margin added to an image-based high-risk volume were shown to reduce the size of the planning target volume when compared to the conventional planning target volume (mean 35%, range 18–46%). It is important to note that an isotropic CTV margin of 2.5 cm for the conventional radiotherapy planning was used in this study. A similar approach by Berberat et al. revealed a trend towards volume reduction using DTI; however, significance was not reached [111]. Trip et al. evaluated coverage of the recurrence volumes by DTI-based CTVs in 40 glioblastoma patients that received standard treatment with CTV margins of 2 cm or less [112]. A slightly worse coverage of the recurrence volumes by the DTI-based CTVs was observed, with central recurrences in particular being covered less. The ability of DTI to predict locations of distant recurrence was explored by Witulla et al., who saw a connection between fiber tracking and the distant recurrence volume in only 1 out of 7 patients [113].

An overview of the different biomarkers and corresponding advanced MR techniques is given in Table 1.

**Table 1:** An overview of the advanced MR biomarkers that have potential to visualize glioblastoma infiltration and predict regions of future recurrence

Biomarker	Advanced MR technique	Physiological process	Reported time of observed alterations seen prior to relapse
Kinetic parameters and CBV	DCE	Vascular leakiness	Not reported
CBF	ASL	Angiogenesis	Not reported
rCBV	DSC	Angiogenesis	4–9 months [46, 48]
VSI	DSC (gradient and spin echo acquisition)	Vasodilatation	1 month [46]
CMRO_2_	qBOLD and ASL	Oxygen consumption	6 months [46]
Amide concentration	APT CEST	Protein concentration	Not reported
d-Glucose	DGE	Glucose uptake	Not reported
Cho/NAA	MRS	Metabolism	4–5 months [93]
ADC	DWI	Cellularity	3.0–3.2 months [104, 105]
Anisotropic diffusion	DTI	Invasiveness	Not reported

## Discussion

This literature review explored the potential of advanced MR imaging to visualize microscopic tumour infiltration and predict future areas of relapse in patients with glioblastoma. Studies using various techniques that (indirectly) visualize tumour growth/metabolism, vascularization, and hypoxia show that advanced MR biomarkers can precede morphologic changes on conventional MR imaging and could be the initial step towards personalization of radiotherapy planning.

Although studies on multiple techniques have shown promising results, the integration of advanced MR imaging with radiotherapy planning in clinical practice remains limited. Careful consideration is taken regarding the introduction of these techniques into clinical practice, as it remains challenging how to incorporate the additional information resulting in either improved local tumour control or reduced radiation-induced toxicity.

Hypoxia and oxygen metabolism showed to be significantly altered approximately 6 months prior to tumour progression being visible on conventional MR imaging [46]. Including this biomarker in radiotherapy planning can provide early information on future sites of relapse compared to other biomarkers reviewed in this paper. The oxygen tension and CMRO_2_ show a turning point at 90 and 60 days before recurrence, respectively. This switch could be an indication of the Warburg effect, in which aerobic glycolysis becomes dominant and cell proliferation is upregulated. This is in concordance with findings of DWI trials that evaluated progression-free survival; significant hypercellularity was reported at a median of 3.0 or 3.2 months prior to recurrence [103, 104]. As a switch is observed during the course of tumour development and radiotherapy planning is performed on a single time-point, sole information on CMRO_2_ or oxygen tension can result in overlooking potential sites of relapse. Depending on the time of acquisition, the oxygen consumption could appear normal after the Warburg effect has become dominant.

A multiparametric approach, where multiple biomarkers were included, was shown to be superior to single-parameter predictive models [47, 114, 115]. Inclusion of biomarkers of perfusion-weighted imaging in addition to oxygenation could allow for a better understanding of the pathological processes in a region. Research on ASL as a technique to visualize tumour infiltration has remained limited. Analysis of the recurrence site showed that regions with increased perfusion acquired with DCE-MR imaging (and hypercellularity acquired with DWI) are likely to progress; however, the majority of failures were seen to occur at sites that did not display alterations on these MR techniques [37]. An increase in microvascular density can be seen 4 months before radiological recurrence, indicating upregulation of hypoxia-induced angiogenesis. In the early stages of angiogenesis, it is believed that angiogenesis is dominated by the formation of smaller vessels, whereas the later stages show transformation of these vessels into larger lumen vessels [46]. Including both rCBV, which correlates with microvascular density, and VSI with DSC can therefore provide complete information on the angiogenic process that occurs in tumour progression.

Combining perfusion and oxygen biomarkers allows for early indication of future sites of relapse while preventing misinterpretation due to the Warburg effect. A region with hypoxia or increased CMRO_2_ without altered perfusion can indicate vessel co-option or a temporary avascular state, which is predictive for future upregulation of angiogenesis and relapse. When a region shows increased rCBV or VSI with normal CMRO_2_, a switch from an infiltrative phenotype to a proliferative phenotype could have occurred [46]. In this case, angiogenesis is upregulated and aerobic glycolysis is dominant indicating rapid cell proliferation and hence tumour growth.

In a preclinical study, Baker et al. showed that, in some cases, vessel co-option can persist through to later stages, with the absence of the switch to angiogenesis for tumour vascularization [116]. As vessel co-option is not measurable through rCBV or vessel size, another biomarker might be needed to prevent misinterpretation of oxygen biomarkers. Tumour growth and metabolism can be directly or indirectly measured using various techniques. The Cho/NAA ratio was shown to correlate accurately with tumour infiltration; however, the heterogeneous nature of glioblastomas accompanied by a relatively poor signal-to-noise ratio and large voxel size of multi-voxel MRS makes this technique less suitable for delineation. The presence of (micro)necrosis within a voxel could result in a decreased Cho/NAA ratio. Techniques utilizing high resolution multi-voxel MRS have been explored; however, an acquisition time of 19 min was required. For both phosphorous MRS and DGE-MR imaging, further research should be conducted on the translation of the techniques to clinical field strengths. Measurements of hypercellularity with ADC mapping has been explored, showing significant hypercellularity to precede future tumour recurrence by 3 months. White matter tracking and the inclusion of DTI information for CTV delineation showed rather disappointing results, but might be useful in a small subgroup that is prone to develop distant progression. Although CEST imaging is a relatively novel technique and the application of the technique in large controlled trials is not yet explored, the studies presented in this review show promising results. Significant differences on both APT and NOE CEST imaging between groups that developed early progression and late progression indicate the predictability of this technique for future sites of relapse and should be further investigated. An additional benefit of CEST for radiotherapy is its ability to distinguish true tumour progression from pseudo-progression, which is a post-radiotherapy phenomenon where damage to epithelial cells and increased inflammation present itself similar to tumour progression on conventional MR imaging [72, 81]. DWI is well established in clinical practice and thus a safe option, yet, within radiotherapy, CEST is a promising technique that should be further investigated. Since both APT and NOE contrasts have shown potential, CEST acquisition schemes in which both contrasts can be assessed should have the preference in future studies.

It is likely that a combination of advanced MR techniques will be required for optimal CTV delineation, but which parameters should or should not be included is an emerging and active field of research. The currently more established biomarkers, coming from widely available acquisitions and having software packages available for analysis, such as rCBV from DSC-MR imaging and ADC from DWI, are to be improved upon for CTV delineation via addition of more elaborate or novel biomarkers. Although DTI is a technique more widespread available already and infiltration along white matter tracts can provide valuable information, it might not be the most suitable technique for CTV delineation. For adaptation in clinical practice, additional research on the more novel MR techniques and their corresponding biomarkers is essential; however, these techniques could eventually be the missing piece of the puzzle. For example, CEST is becoming more and more available with the research community reaching consensus on acquisition and analysis [117] and qBOLD imaging showed promising results compared to other biomarkers (including rCBV and ADC) [46]. Biomarkers ultimately included for CTV delineation should convey complementary information, be sensitive for glioblastoma pathophysiology, and have sufficient reproducibility and repeatability to determine cut-off values for multi-center, multi-vendor use.

## Conclusion

The potential of advanced MR imaging for the visualization of microscopic tumour infiltration and prediction of future relapse sites has been presented. Including biomarkers that provide information on oxygen consumption (CMRO_2_ or oxygen tension), angiogenesis (rCBV and VSI), hypercellularity (ADC), and protein concentration (APT) in radiotherapy planning of glioblastoma may result in more accurate CTV delineation, improved local tumour control, and/or reduced radiation-induced toxicity.
